# Brain-heart axis - Review Article

**Published:** 2015

**Authors:** MM Manea, M Comsa, A Minca, D Dragos, Constantin Popa

**Affiliations:** * Neurologic Clinic, National Institute of Neurology and Cerebrovascular Diseases, Bucharest, Romania; “Carol Davila” University of Medicine and Pharmacy, Bucharest, Romania; ** Internal Medicine Clinic; University Emergency Hospital, Bucharest, Romania; “Carol Davila” University of Medicine and Pharmacy, Bucharest, Romania; *** Nephrology Department, University Emergency Hospital, Bucharest, Romania; “Carol Davila” University of Medicine and Pharmacy, Bucharest, Romania; **** “Carol Davila” University of Medicine and Pharmacy, Bucharest, Romania; Romanian Society of Stroke; Neurology Department, National Institute of Neurology and Cerebrovascular Diseases, Bucharest, Romania

**Keywords:** stroke, heart, elevated cardiac enzymes, sudden death

## Abstract

There has been a large confirmation over the last decades that stroke may produce cardiac changes (echocardiographic, electrocardiographic, enzymatic). In ischemic stroke, systolic dysfunction is associated with a high risk of mortality during hospitalization. A recent study demonstrated that cardiac diastolic dysfunction could also accompany acute stroke besides the systolic dysfunction already pointed out by previous studies, being a predictive marker of acute cerebrovascular events. Increased sympathetic activity is contributory, inducing a reversible cardiac myocyte damage and cardiac enzyme surges. Some of the most frequent electrocardiographic abnormalities in stroke are ST segment abnormalities and various tachyarrhythmias (especially atrial fibrillation) and bradyarrhythmias. One can infer the importance of careful and continuous electrocardiographic monitoring of the stroke patient in order to identify these quite frequent electrocardiographic alterations, as it is well known that death due to cardiac arrhythmias is common among acute stroke patients. In order to increase the diagnostic yield, a high level of NTproBNP (N-terminal of the prohormone brain natriuretic peptide) may be used as a discriminant for the patients with a higher probability of cardiac arrhythmias and mortality at presentation, during hospitalization and on the long term. In such patients, cardiac monitoring techniques are more likely to reveal abnormalities. A high BNP level may have potentially important management implications as it may signal a worse prognosis and may prompt the undertaking of certain therapeutic measures. This review summarizes the possible pathological mechanisms of heart-brain connections and their clinical and therapeutical implications.

**Abbreviations:** AF = atrial fibrillation, ECG = electrocardiography, HRV = heart rate variability, cTn = cardiac troponin, SAH = subarachnoid hemorrhage, CK-MB = creatine kinase-MB, BNP = brain natriuretic peptide, NT-proBNP = N-terminal of the prohormone brain natriuretic peptide, ANP = atrial natriuretic peptide, mRS = modified Rankin Scale, NIHSS = the National Institutes of Health Stroke Scale.

## Introduction

Stroke is one of the most important causes of mortality and morbidity in industrialized countries. In Europe, it is the second mortality cause, accounting for approximately 10% of all death causes in men and 15% in women [**1**]. More than half of the stroke patients are left with a major disability [**2**,**3**]. Approximately 85% of strokes are ischaemic, the remainder 15% being haemorrhagic [**4**]. The most frequent causes of ischaemic stroke are atherosclerosis - 50%, [**5**] (- P262), cardioembolism - 20%, [**5**] (- P260) and microinfarcts (lacunar strokes) - 25% [**5**] (- P270) [**3**]. Rare causes (responsible for 5% of cases) are arterial dissection and patent foramen ovale [**5**] (- P355-358).

**Neuro-heart disease**

In the last decades, the study of the connection between brain and heart has grown ever more important, the concept of neurocardiology being born. Literature revealed that the most frequent cause of the stroke is cardiac arrhythmia, most often AF. Beside the risk of cerebral embolism, AF also represents a risk factor for hippocampal atrophy and for cognitive dysfunction [**6**]. Cerebrovascular diseases can induce electrocardiographic abnormalities and a variety of cardiac arrhythmias that can sometimes cause sudden death. These findings have major clinical and therapeutical implications in patients with cerebrovascular events [**6**]. 

The first findings about the neural control over the body as a whole were made about 200 years ago by Magendie [**7**].

The anterior cingulate cortex, amygdala, parabrachial nucleus, hypothalamus, periaqueductal grey matter, the anterior insula and some areas of the medulla have an important role in modulating the cardiac function. Through the sympathetic and parasympathetic nervous system, these cerebral structures are involved in cardiac activity (modulating the force of contraction and heart rate), in the responses to stressful and emotional events and in the homeostatic reflexes [**8**]. When injured, the right insular cortex may be the origin of sympathetic discharges leading to the cardiac alterations typical for stroke, whereas parasympathetic activity may increase as a consequence of left insular cortex damage [**9**]. Our current knowledge in neuroanatomy and neurophysiology cannot entirely explain either these phenomena, or the lateralization mechanisms [**8**]. The “laterality hypothesis” can also be applied to emotions: negative emotions originate mainly in the right hemisphere, while positive emotions arise especially in the left hemisphere [**10**]. A similar lateralization of the autonomic activity exists in the heart (right- and left-sided nerves being distributed to the right and left ventricle respectively) [**10**]. 

**Laboratory animal studies**

Many of these studies have shown that some neurological disease can induce anatomical and functional cardiac alterations. 

The first report in this field was Openheimer’s and his coworkers’. They noted that the stimulation of a cortical region could induce cardiac rate alteration, without affecting other parameters. According to the laboratory studies, the insular cortex plays a crucial role in the management of the heart’s chronotropic activity. While tachycardia occurs by stimulating the rostral posterior insula, the stimulation of the caudal region of the posterior insula evokes brachycardia. It was shown that these cardiac rhythm variations could be stopped by administering atenolol, but not atropine, thereby proving the influence of the sympathetic nervous system over cardiac activity [**11**]. 

The tachycardia induced by stimulating the rostral insular cortex is exclusively accompanied by a noradrenaline (but not adrenaline) surge. By contrast, no change in the catecholamines level is produced by stimulating the caudal region of the posterior insula. The results demonstrate that tachycardia depends on the sympathetic nervous system, while brachycardia on the parasympathetic nervous system [**12**]. 

In support of these theories, Melville described that the stimulation of the anterior hypothalamus in cats produces a burst of parasympathetic activity while ensuing bradycardia. In contrast, the stimulation of the lateral hypothalamus increases heart rate and alters the ECG [**13**]. 

Furthermore, that pathological stimulation of the insular cortex by an epileptic seizure, a stroke, or a severe emotional stress may provoke ECG abnormalities, cardiac arrhythmias, and even sudden death [**14**,**15**]. 

**Autonomic nervous system**

Autonomic systems (sympathetic and parasympathetic structures, suprachiasmatic nucleus, higher nervous centres, including some of those involved in depressive and aggressive behavior) play an important role in the control of heart rate variability (HRV), which is an important risk factor for atherosclerosis, arrhythmias, heart failure, myocardial infarction, and sudden cardiac death [**6**].

While an increased sympathetic tone determines high levels of catecholamines, cortisol, serotonin, renin, angiotensin, aldosterone, and free radicals, enhanced parasympathetic activity is correlated with high levels of dopamine, acetylcholine, endorphins, nitric oxide, Q10 coenzyme, and other protective substances [**6**]. Any agent that can increase brain acetylcholine level can protect the suprachiasmatic nucleus, keeping in check the sympathetic activity and thereby mitigating the risk of myocardial infarction and sudden death [**6**].

Laboratory studies have shown that, in stroke, catecholamines are discharged in large quantities, resulting in cardiac dysfunction and an increase in cTn concentration. High levels of catecholamines determine the release of intracellular calcium and reversible cardiac myocyte damage [**16**]. 

To back up this affirmation, another study noted that cardiac systolic dysfunction is correlated with surprisingly normal myocardial perfusion, but increased sympathetic activity [**17**]. 

Enhanced parasympathetic activity has a protective role for the ventricles, while sympathetic stimulation produces arrhythmia. In contrast, in prolonged QT syndrome and Brugada syndrome, increased parasympathetic activity produces arrhythmias, although it is usually very difficult to tell enhanced vagal activity from decreased sympathetic activity [**18**].

Parasympathetic activity is increased by the stimulation of the posteroinferior parts of left ventricle, while the stimulation of the anterior left ventricle enhances the efferent sympathetic tone [**10**]. 

Clinical and experimental data suggest a correlation between sudden cardiac death and mental stress: interrupting the communication between the frontal cortex and brain stem pathways preventing stress-induced ventricular fibrillation, while vagal stimulation plays a protective role from stress-induced cardiac arrhythmias. Conversely, ventricular fibrillation is precipitated in long-QT syndrome by emotional arousal [**10**]. 

**Cardiac changes**

**Electrocardiographic changes and cardiac arrhythmias**

There has been a large confirmation over the last decades that stroke may produce cardiac changes [**19**]. The first to describe electrocardiographic alterations in stroke were Bodechtel and Aschnebrenner (1938) and Byer& Co (in 1947). [**20**]. 

These electrocardiographic (ECG) alterations in stroke can appear in the context of a preexistent cardiac disease, but also in its absence, in which case they have a neurologic origin [**20**,**21**]. The incidence of these manifestations is higher in subarachnoid hemorrhage (SAH) (60-70% of patients) and its complications [**20**], than in ischemic stroke (15-20%) [**19**]. Cardiac arrhythmias are more frequent in right cerebral hemisphere strokes compared to those afflicting the left cerebral hemisphere [**22**]. 

Some of the most frequent electrocardiographic abnormalities in stroke are: ST segment abnormalities, negative T waves, U waves [**16**,**19**,**23**], left axis deviation [**19**], prolonged QT [**19**], atrial fibrillation (AF)/ atrial flutter, sinus tachycardia, ventricular tachycardia, atrial and ventricular premature complexes, bradyarrhythmias (sinus-node dysfunction, 2nd and 3rd degree heart block) [**24**]. 

Atrial fibrillation is the most common tachyarrhythmia in acute stroke and is associated with an increased risk of systemic thromboembolism [**23**]. 

Rarely, other tachyarrhythmias, such as atrial tachycardia, can occur. Tachyarrhythmias can increase the extent and/ or severity of the cerebral damage during a stroke or in its aftermath (by lowering the cerebral perfusion) and can also increase the risk of ischemic cardiac lesions in patients with a history of heart disease [**23**]. A long QT can precipitate a ventricular polymorphic tachycardia and sudden cardiac death [**23**]. One cannot overemphasize the importance of careful and continuous ECG monitoring of the stroke patient in order to identify these quite frequent electrocardiographic alterations, as it is well known that death due to cardiac arrhythmias is common among acute stroke patients [**23**,**25**]. 

**Echocardiographic changes in stroke**

**Systolic dysfunction**

Cardiac systolic dysfunction has been noted in all three types of stroke. Neurogenic stunned myocardium is not infrequent after SAH: between 2% to 30% of the patients are afflicted [**16**]. Ventricular dysfunction is characterized by hypokinetic wall-motion changes on echocardiography. Previous studies demonstrated the absence of perfusion defects on radionuclide studies and of vascular lesions on coronary angiography in SAH cases. The transient character of the above-mentioned wall motion changes further proves their neurogenic nature [**16**]. 

Systolic dysfunction on echocardiography appears in 13-29% of the cases of ischemic stroke. These changes are associated with a high risk of mortality during hospitalization (and so are cardiac troponin (cTn) elevation and AF) [**26**].

**Diastolic dysfunction**

A recent study demonstrated that cardiac diastolic dysfunction could also accompany acute stroke [**27**], besides the systolic dysfunction already pointed out by previous studies [**16**]. 

Cardiac diastolic dysfunction results in elevated left ventricular end diastolic pressure and sympathetic arousal, leading to injury of endothelial cells and consequent hypercoagulability [**27**]. It has been demonstrated that the diastolic dysfunction (echocardiographically reflected in the E/ A ratio) is a predictive marker of acute stroke [**27**]. 

**Serum enzymatic changes in stroke**

In 1979, Hachinski & Co described high levels of cardiac enzymes in acute stroke [**28**]. Later studies endorsed this original finding and described in detail the involvement of the sympathetic nervous system in these biohumoral alterations [**29**]. 

**Myoglobin**

A recent prospective study has shown that myoglobin level (a sensitive but not specific indicator for myocardial infarction) is higher in patients with ischemic stroke and a critical state than in those in a better condition [**36**]. Although there is no unanimity regarding these correlations, Guerrero et al. showed that myoglobin level was not related to the stroke’s severity [**31**]. 

**Creatine kinase-MB**

Creatine kinase-MB (CK-MB) does not hold the upper hand in specificity over the other cardiac markers, as it may increase in situations other than myocardial infarction, such as toxin or drug exposure, or in renal insufficiency. Although high CK-MB levels have been proposed as a humoral marker for stroke-related myocardial damage, it seems that CK-MB increases in some cases of cerebral infarction in the absence of a provable acute myocardial infarction. Troponin T is more sensitive and specific compared to CK-MB in detecting slight myocardial injury, and it has been shown not to increase after cerebral infarction. The fact that a normal cTn can coexist with a high CK-MB underlines that CK-MB should not be considered a biochemical marker for cardiac myocytolysis, as its elevation in acute stroke might not be of cardiac origin [**29**,**32**]. 

Clinical and experimental data suggest that cardiac abnormalities in stroke are the result of increased sympathetic activity secondary to insular cortical damage. Similarly to other studies, Butcher et al noted increased CK-MB in some stroke patients but, unlike previous studies, no correlation was found between the high CK-MB levels and sympathetic activity. The authors suggested that the origin of the elevated CK-MB might be non-cardiac in some patients and cardiac in others. An abnormal sympathetic activity may be one of the factors leading to cardiac enzyme changes [**29**]. 

**Troponin**

Myocardial infarction is the consequence of prolonged cardiac ischemia. cTn emerged as the most specific marker for cardiac lesions. Unfortunately, it is not only the most sensitive (even minimal lesions lead to increased cTn levels), but also too sensitive, as it fails both to differentiate necrosis from mere lesion (without actual cell death), and to point out the underlying condition. Increased cTn levels can occur also in various other conditions (than acute myocardial infarction): stroke, acute myocarditis, acute pericarditis (with accompanying epicarditis and superficial myocarditis), sepsis, pulmonary embolism, heart failure, renal insufficiency etc. [**16**,**33**].

Meta-analyses have shown that more ECG changes are to be expected in patients with stroke and high level of cTn than in those with normal levels of cTn. Similarly, the risk of death was related to the cTn level, being higher in patients with increased cTn [**16**,**34**-**38**].

A cardiovascular disease (such as heart failure or atrial fibrillation) in the history of the patient may be the reason for the increased level of cTn. However, what about patients with no known cardiac disease? Radionuclide studies comparing patients with high levels of cTn with those with normal levels of cTn and no proof of cardiac ischemia have shown no difference in the myocardial perfusion abnormalities found in the two categories of patients [**16**]. In conclusion, in the cases of ischemic stroke but without a history of ischemic heart disease, cTn is rarely increased, and when it is, its high levels are attributable to renal or cardiac insufficiency, rather than to myocardial infarction. Nevertheless, as previously mentioned, the prognosis of these patients is darkened by a higher risk of death in the next two years [**38**].

**Natriuretic peptide**

There are 3 types of natriuretic peptides: BNP (brain natriuretic peptide, mainly secreted by the ventricles), ANP (atrial natriuretic peptide, secreted in the right atrium), and C type of natriuretic peptide (the secretion of which is induced by the inflammation and lesion of the vascular endothelium) [**39**].

BNP is a neurohormone primarily secreted in the ventricles in response to volume and pressure overload [**40**]. Nonetheless, as its name implies, this neurohormone is also secreted in the brain [thalamus, hypothalamus, cerebral cortex, brain stem (pons) and cerebellum] [**41**,**42**]. Its vasodilator and natriuretic properties rely on direct effects on the renal vessels and tubules, corroborated by neurohormonal actions including the inhibition of the sympathetic and renin-angiotensin-aldosterone systems [**40**]. 

BNP is used to evaluate cardiac failure, but its increase in acute stroke can be not only the result of the ventricular systolic dysfunction, but also of enhanced sympathetic activity [**40**]. 

Recent studies showed that the BNP level is higher in patients with paroxysmal AF. An increased level of BNP may be a harbinger of AF [**43**-**47**]. 

On the other hand, plasma BNP levels are higher in patients with larger ischemic strokes, higher CHADS2 scores and higher mRS (modified Rankin Scale) scores [**48**]. 

A first study of Nigro et al. evaluated the possibility of a connection between increased BNP or cTn levels and the risk for stroke recurrence, but failed to establish such a connection [**49**].

**Prognostic**

It is difficult to estimate the prognosis in stroke patients. A study on 122 patients with acute ischemic stroke attempted to find a correlation between BNP level and NIHSS scale (the National Institutes of Health Stroke Scale). The higher mortality in patients which increases in both BNP values, and NIHHS scores at admission, endorsed the conclusion that the combination of elevated BNP and NIHHS score should be considered an indicator of grim prognosis in stroke patients [**50**].

As data on this subject are contradictory, García-Berrocoso endeavored to cast some light upon the putative correlation between BNP or NT-proBNP, (N-terminal of the prohormone brain natriuretic peptide) values and mortality in the aftermath of a stroke. Using MEDLINE and EMBASE databases, the authors conducted a search for relevant articles published until October 2012. The ensuing meta-analysis proved BNP to be a predictive marker for the post-stroke mortality, independent of the NIHHS score and demographic factors (sex and age) [**51**]. 

An increased natriuretic BNP level in acute stroke is a predictor of mortality at presentation, during hospitalization, and after discharge on the long term [**49**,**52**]. 

In addition, positive cTnI and ECG abnormalities [**25**] at admission were related to a bad prognosis in acute stroke patients [**36**,**37**]. 

## Discussion

In 20 percent of the cases, ischemic stroke is caused by a cardiac disease, the major culprit being AF, which increases the risk of cerebrovascular events by about 5 times [**3**,**53**] inducing approximately 12.500 cerebrovascular infarctions per year in patients with cardioembolism [**3**,**54**]. The correct treatment for AF can reduce this number by about 4500 cases, and can prevent about 3000 stroke-related deaths annually. In these cases, oral anticoagulation is recommended for the secondary prevention [**3**,**54**].

In cryptogenic ischemic strokes, the cause may remain obscure (it is commonly surmised that paroxysmal AF is at the origin of most such cases) [**55**], therefore, no specific medication is warranted [**40**].

It has been demonstrated that higher BNP levels correlate with AF (both chronic and paroxysmal) [**43**]. Cardiac-monitoring strategies (Holter, subcutaneously implanted monitors) can detect AF and other arrhythmias, but only in a small percentage of patients. In order to increase the diagnostic yield, a high level of NTproBNP may be used as a discriminant for the patients with a higher probability of cardiac arrhythmias and therefore of positive results on cardiac monitoring techniques [**43**]. 

A high BNP level may have potentially (very) important management implications as it may signal a worse prognosis and may prompt the undertaking of certain therapeutic measures [**40**,**43**,**56**,**57**]. 

The explanation for the unpredictable and sudden cardiac changes accompanying acute stroke probably lies in the interplay between brain and heart, highlighting the need for a better understanding of and possibly, for new approaches to this matter, which may lead to novel therapeutics aimed at preventing sudden death in cerebral infarction patients [**58**]. 

**Acknowledgements**

I would like to express the deepest appreciation to Academician Professor Constantin Popa, who has shown the attitude and the substance of a genius: he continually and persuasively conveyed a spirit of adventure concerning research and scholarship, and an excitement about teaching. Without his supervision and constant help, this review would not have been possible. Also my thanks and appreciations go to my colleague in developing the project, and people who have willingly helped me out with their abilities.

**Disclosures**

None.
